# Post-COVID trends in hospital financial performance: updated data from California paint an improved but challenging picture for hospitals and commercially insured patients

**DOI:** 10.1093/haschl/qxad039

**Published:** 2023-08-24

**Authors:** Glenn Melnick, Susan Maerki

**Affiliations:** University of Southern California, CA, United States; Independent Health Consultant San Francisco CA 94122, United States

**Keywords:** hospital financial status, hospital prices

## Abstract

The COVID-19 pandemic caused major disruptions to the operation and financing of US acute care hospitals. Previous research has documented early effects of the COVID pandemic on hospital financial performance. This paper updates the literature with current data on utilization and financial performance for a large sample of California hospitals covering the period 2017 through the end of 2022 and the first quarter of 2023. The data show that, while hospital overall utilization has largely returned to pre-COVID levels, patient mix has changed and financial performance still lags. Hospital net income margins remain below pre-COVID levels which could trigger price increases to commercially insured patients to offset continuing post-COVID financial shortfalls.

## Lay Summary

COVID-19 created substantial disruptions and stresses to our health care system, including hospitals, and as such, it is critical to understand how hospital financial performance continues to be affected by these challenges. This study analyzes current data (through quarter [Q] 1 of 2023) to assess evolving financial trends affecting hospitals. The data show that hospitals weathered the initial impact in 2020 and 2021 but encountered challenging financial conditions in 2022 as net income from both operating and nonoperating sources fell dramatically in the face of a sustained increase in operating costs and decreasing revenue from nonoperating sources. Pressures on net income from operations persisted through Q1 of 2023, although nonoperating income has rebounded. Importantly, the data show that trends vary substantially across hospitals and that there is a large subset of hospitals that remain under severe financial pressure and may require assistance from policymakers to sustain operations until their financial positions are stabilized.

## Teaser

The COVID-19 pandemic caused major disruptions to the operation and financing of US acute care hospitals. This paper updates the literature with current data to document the evolving financial status and challenges for US hospitals, post-COVID. While financial performance appears to be improving, many hospitals have not fully recovered from the pandemic and face headwinds that will affect both hospitals and patients in the years to come.

## Introduction

In March 2020, most acute care hospitals across the United States began postponing elective admissions and nonurgent care due to a declaration of a public health emergency and an expected surge of patients with COVID-19. Across the country, hospitals expanded inpatient capacity to repurpose space and to procure additional equipment, supplies, and staff. Meanwhile, a significant number of patients began to forgo care as economic activity and travel dropped precipitously. These developments had immediate impacts on hospital utilization, costs, and revenue, with inpatient admissions, outpatient services, and emergency department visits falling rapidly and substantially. Previous research has documented some of the financial impacts of the pandemic on the financial performance of hospitals in the United States.^1^ With the COVID-19 pandemic largely behind us, the hope is that our health care system and other aspects of our lives would return to normal. This paper updates the literature with more current data on utilization and financial performance for a large sample of hospitals from California covering the period 2017 through the end of 2022 and the first quarter of 2023.

## Data and methods

(Please see the Supplemental Technical Appendix for a detailed description of Data and methods.)

### Data

California hospitals report financial and utilization data on both a quarterly and annual basis. The quarterly data are filed within 90 days of the close of each quarter, providing data on a more real-time basis. Our study uses the quarterly data and our sample acute care hospitals that reported data to the California Office of Health Care Access and Information (formerly the Office of Statewide Health Planning and Development).^2^ Because we are relying on financial performance measures using quarterly data that are reported with the shortest lag time, we compared historical quarterly and annual data covering the same time periods and found that, for the utilization and financial performance measures, the revisions to quarterly data, when compared with annual audited data, were quite limited both on an aggregate basis at the hospital level (although hospital-level adjustments had a wider range).

### Statistical methods

Our methods are straightforward in that we sum individual hospital-reported totals for each utilization and financial performance measure across all hospitals in the sample for each quarter or 4 quarters combined for each year. For one analysis (Figure 3), we calculate hospital net income margins for each hospital and summarize the distribution across all reporting hospitals for different percentile cut points. Our figures present simple descriptive statistics using these aggregated data and hospital level data.

## Results

### Hospital utilization: total hospital volume has returned to pre-COVID levels, but with changes

**Figure 1.**
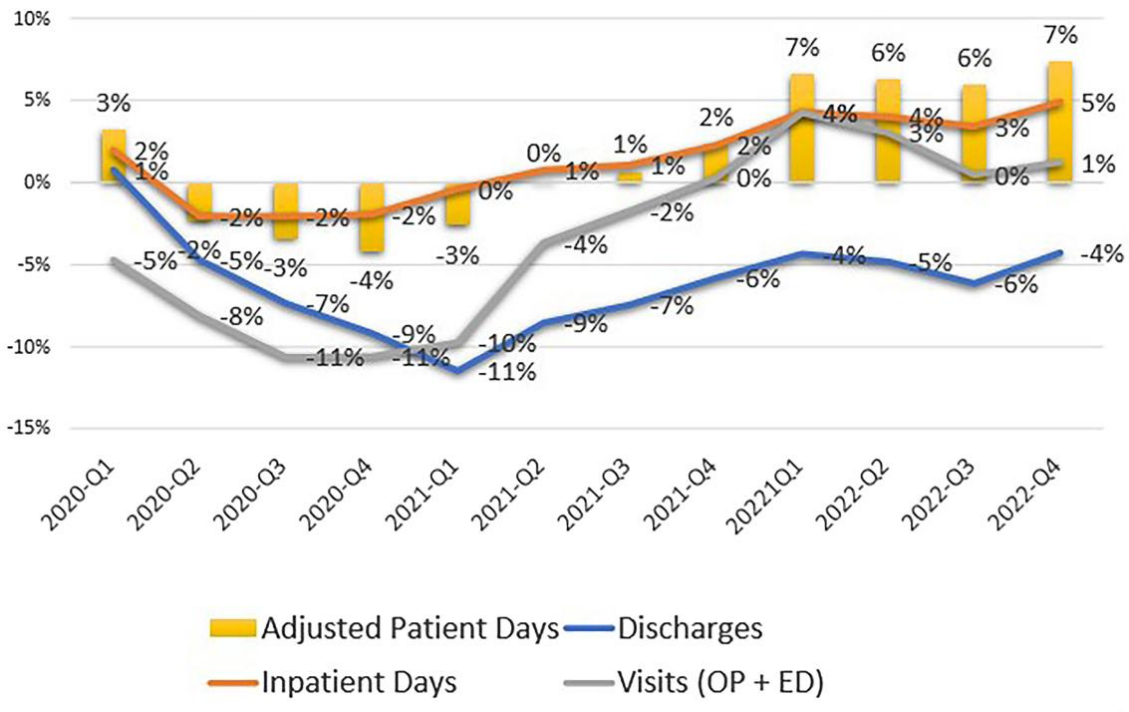
California hospital utilization 2020–2022. Inpatient discharges, inpatient days, outpatient visits, and adjusted patient days compared to 2019. Abbreviations: ED, emergency department; OP, outpatient; Q, quarter

Figure 1 shows that total hospital volume (measured by total adjusted patient days [which combines inpatient plus outpatient services based on gross charges per day]) rebounded to pre-COVID levels by Q2 of 2021, continued to rebound, and, as of Q4 of 2022, exceeded pre-pandemic levels by 7%. However, the mix and nature of hospital services have changed since the onset of COVID-19. The number of inpatients admitted to hospitals is still approximately 4% below pre-pandemic levels and total outpatient volume (including outpatient and emergency department visits) are slightly above 2019 levels.

**Figure 2.**
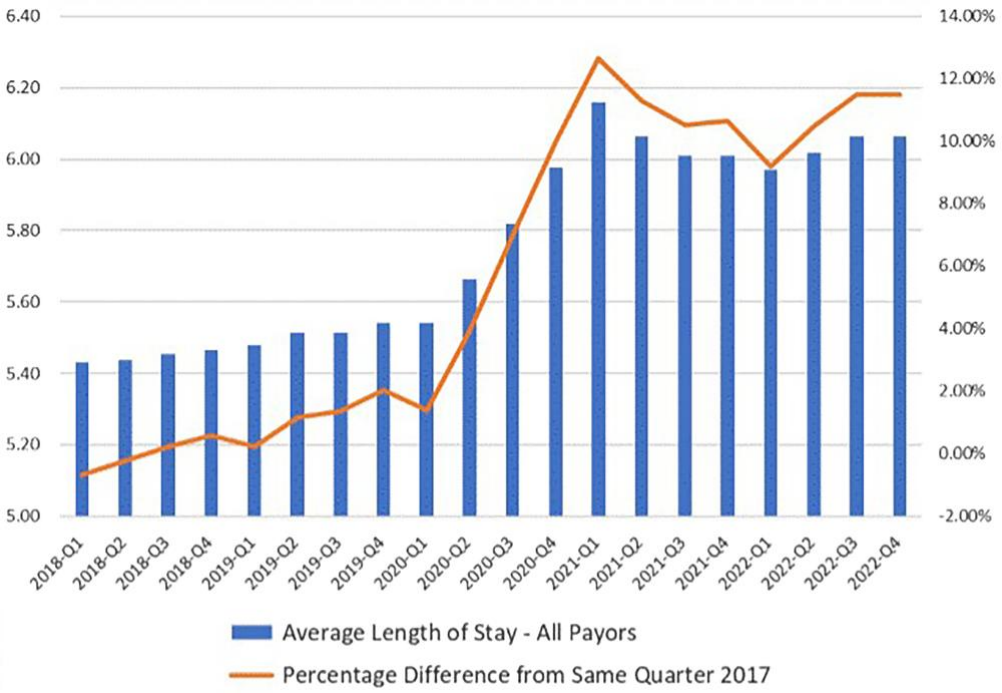
California hospital utilization 2018–2022. Inpatient average length of stay. Abbreviation: Q, quarter.

Figure 2 shows the trend in average length of stay (ALOS) of patients admitted to California hospitals both before and after the COVID-19 pandemic. The ALOS had been relatively stable for the 8 quarters before Q1 of 2020, growing slightly (1.8%) between Q1 of 2018 and Q4 of 2019. In Q2 of 2020, ALOS began to lengthen and accelerated through 2020 and Q1 of 2021. This likely reflects the first major surge of patients with COVID-19 plus a changing mix of other admitted patients that did not include elective procedures or nonurgent conditions and with more complicated medical conditions requiring additional resources and time in the hospital. Importantly, this longer ALOS trend has continued through 2021 and 2022, suggesting a possible permanent shift in hospital case mix for patients admitted to California hospitals. At the same time, there have been reports of staff shortages in postdischarge facilities, which has delayed discharge of patients from acute care hospitals, possibly contributing to longer lengths of stay.

### Hospital financial performance: hospitals face continuing financial pressure as they emerge from the COVID-19 pandemic

As shown in Table 1, hospital financial performance pre-COVID trends were positive and relatively stable before being interrupted by the COVID-19 pandemic. In the 3 years preceding COVID-19 (2017–2019), California hospitals averaged nearly $5 billion in net income from operations and over $3 billion in net income from nonoperating sources, for an average of $8.4 billion in total net income (pre-tax). In 2020, total net income dropped to $5.2 billion, driven largely by reduced income from operations. The 2020 operating result was buoyed by government COVID-19–related subsidies and significantly offset by increased nonoperating income, largely from outside investment income.

**Table 1.** California hospital financial performance, 2017–2022: components of hospital net income, 2017–2022 (4 quarters summed) and 2023 Q1.

	2017	2018	2019	Average 2017-2019	2020	2021	2022	2023-Q1
Net income—operating ($ billions)	4.75	5.64	4.00	4.79	−0.22	2.50	0.10	−0.20
Net income—nonoperating ($ billions)	3.48	1.47	5.93	3.63	5.41	6.71	0.11	1.86
Net income—total (pre-tax, $ billions)	8.23	7.10	9.93	8.42	5.19	9.20	0.21	1.66

Abbreviation: Q, quarter.

Total net income increased in 2021 to more than $9 billion, a result of $2.5 billion in positive net income from operations, as volume rebounded to above pre-COVID levels and a second year of substantial net nonoperating revenue. This positive trend reversed in 2022: net income from both operating and nonoperating sources virtually disappeared in 2022 and, as a result, total net income barely exceeded break-even in 2022 at $207 million. The data for Q1 of 2023 show a significant rebound in nonoperating net income, returning to pre-COVID levels—totaling a positive $1.857 billion for Q1 of 2023 (annualized, $7.431 billion). However, operating net income has not recovered to pre-COVID levels and, in fact, remained at negative $200 million (annualized, negative $801 million).

The updated, post-COVID data for 2022 and Q1 of 2023 paint a picture of significant financial challenges to California hospitals’ operations as it appears the COVID-19–driven surge in operating expenses due to staff and supply shortages has continued to ripple through hospital operating budgets despite staff reductions and other cost efficiencies. Many hospitals are now more dependent than ever on nonoperating income, which is largely determined by external forces.^3^

**Figure 3.**
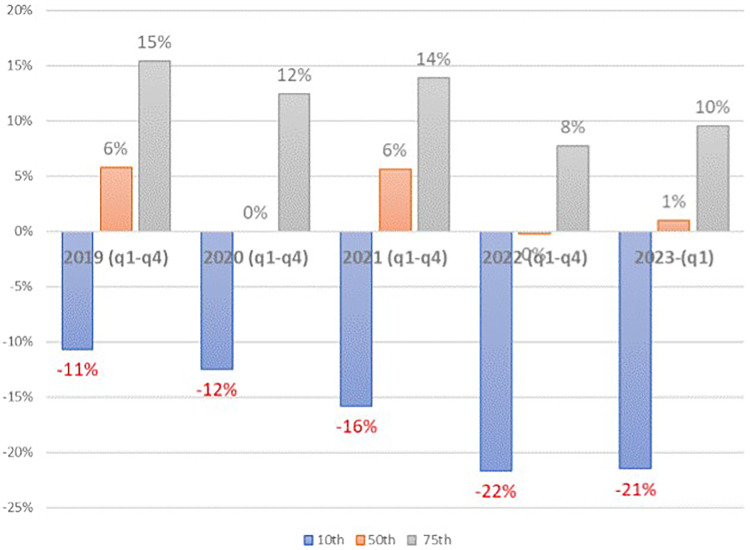
Distribution of hospital total margins (%), 10th, median, and 75th percentile values: 2019–2023 Q1. Abbreviation: Q/q, quarter.

Figure 3 shows the substantial variation in the financial performance of hospitals as measured by the distribution of total net income margins (%, total net income divided by total revenue from all sources) for each year during 2019–2022 and 2023 Q1. As can be seen, trends vary for the different percentile cut points. Median values fell significantly in 2020 but remained positive, due in large part to government COVID-19–related subsidies, rebounded in 2021, and have fallen again well below pre-COVID levels in 2022 and 2023 Q1. The trend for hospitals at the 10th percentile is one of sustained deterioration in total margins, except for a slight improvement in 2023 Q1. The trend for the 75th percentile is the most stable but still shows a significant deterioration in 2022 and Q1 of 2023 compared with pre-COVID levels. As another indicator of the variation in hospital financial performance, the average total margin when data are summed across all hospitals is 4.64%, reflecting the fact that net income margins for larger hospitals are higher than for smaller hospitals.

## Discussion

Overall hospital volume, as measured by total adjusted patient-days, has recovered from the pandemic-related disruptions, along with what may be a permanent shift in the mix of services, including the following: reduced reliance on inpatient admissions, longer lengths of stay per inpatient, and increased utilization of outpatient services. And, while total margins appear to be recovering in Q1 of 2023 (compared with 2022), due in large part to improved outside investment income, operating margins are still lagging. Persistent negative operating margins are explained in part by COVID-19–induced increases in operating expenses that appear to have resulted in permanently increased cost structures and continuing expense inflation above pre-COVID levels. However, within the large sample of hospitals included in this analysis, our data show a significant deterioration in hospital margins in 2022 due to operating losses combined with reductions and losses in nonoperating income. The data for the first quarter of 2023 show a significant improvement in total net income due entirely to nonoperating sources; operating margins remain substantially depressed relative to pre-COVID levels.

### Hospital responses to financial stresses

One of the positive effects of economic pressures in our competitive and dynamic economy is the forced creation of innovative responses following an economic downturn. There are several noteworthy examples from California hospitals of such innovation. Some hospitals began adapting to the changing economic conditions early by both cutting costs and implementing innovative solutions to longer term problems. For example, 1 safety-net hospital implemented a program to solve their nursing shortage and labor cost problem by partnering with nursing schools to offer training to existing staff in order to expand the pipeline of nurses available to their facility in the coming years.^4^ Other hospitals have implemented turnaround plans that include staff reductions (mostly non-nursing staff) and reduced or discontinued services. Many hospitals have announced plans to restructure their operations to achieve efficiencies and lower operating costs, both in California and across the country, including, in some cases, sizeable staff reductions as well as closing selected services within the hospital. Some of these actions likely contributed to the observed improvements in margin in Q1 of 2023.^5^ Hospitals with sufficient cash reserves and other assets have drawn from those holdings and those in multi-hospital systems may have accessed the financial resources of affiliated hospitals and their corporate health care entity.

### Hospital face continued headwinds with limited help from government payers

While 2023 Q1 data suggest a possible trend toward more positive financial outcomes, hospitals continue to face both COVID-19–created and new headwinds. Although there are signs that economywide inflation may be slowing, there are uncertainties regarding rising interest rates, continued employment layoffs, and investment market performance. Shortages in health manpower, drugs, and other supplies contribute to expenses that are still increasing above pre-COVID levels.^6^

Hospital volume and revenue from serving Medicaid patients could be negatively impacted by the Federal Consolidated Appropriations Act of 2023, which set March 31, 2023, as the end date for the Medicaid continuous enrollment. A recent study projected an 18% reduction in Medicaid enrollment (17 million covered lives) across all states between March 2023 and May 2024.^7^ Nearly 3.3 million people in 35 states have already been disenrolled as of July 21, 2023, most due to procedural issues^8^ and California recently reported an estimated 225 000 dis-enrollees among the first 1 million beneficiaries asked to document eligibility.^9^

Reductions in payments from the Medicaid Disproportionate Share Hospital program, enacted as part of the Affordable Care Act with the reasoning that hospitals would have less uncompensated care, as health insurance coverage have been delayed, but if implemented could include cuts of $8 billion for each of the next 4 fiscal years (2024–2027, for a total of $32 billion for all 4 years).^10^

On the plus side for hospitals, annual increases in Medicare payments to hospitals are scheduled to increase but at rates that are likely to be less than hospital operating cost increases.^11^ Payment policies under the Medicare program have a significant impact on all hospitals as the Medicare program is the largest payor for most hospitals in the United States. For example, in April 2023, the Biden Administration proposed a 2.8% bump to inpatient payments to eligible hospitals in the next fiscal year, a $3.3 billion boost for facilities.

### Policymakers recognize that some distressed hospitals may need financial support through targeted subsidies

One important finding from our data relates to substantial variability in financial performance across hospitals. We observed a wide range in total margins across hospitals, with the top performing hospitals showing limited declines in total margins, while those at the low end of the distribution show significant and growing sustained financial strain. Policymakers at the federal and state levels increasingly recognize the need to address the variability in financial stress across hospitals.

The Medicare Payment Advisory Commission’s annual March report to Congress^12^ stated that “General acute care hospitals don’t need a significant increase in 2024 Medicare rates to stay afloat” and recommended that a fiscal year 2024 update to hospital payment rates of current law plus 1% would generally be adequate to maintain for fee-for-service beneficiaries’ access to hospital inpatient and outpatient care but also recognized that its 2024 payment recommendations “may not be sufficient” to sustain some safety-net hospitals with a low number of commercially insured patients and recommended distributing Medicare payments through its Medicare Safety-Net Index, along with adding $2 billion in add-on payments to “help maintain the financial viability of Medicare safety-net hospitals.”

At the state level, Governor Newsom of California recently signed into law a bill that establishes the Distressed Hospital Loan Program (Chapter 6, Statutes of 2023 [AB 112]). The program (funded initially at $150 million and recently increased to $300 million) provides interest-free cashflow loans to not-for-profit and public hospitals in significant financial distress or to governmental entities representing a closed hospital to prevent the closure of or facilitate the reopening of the hospital. Investor-owned hospitals, freestanding psychiatric inpatient hospitals, and hospitals that are part of a system of more than 2 hospitals are not eligible.

## Conclusion

Overall, the data presented here, while limited to California hospitals, are likely representative of hospitals across the United States and paint a picture of a tremendously challenging future. While volume has returned to pre-COVID levels, it appears that case-mix complexity and the unit costs of labor and nonlabor inputs have increased, resulting in a permanently increased cost structure.^13^ At the same time, net income from both operating and nonoperating sources evaporated in 2022. Data covering operating and nonoperating performance for Q1 of 2023 show a significant rebound in net nonoperating revenue, but operating margins are still lagging. Total margins aggregated across all hospitals for Q1 of 2023 reached 4.64%, but operating margins across those hospitals are still negative at −0.56%.

Hospitals, like all organizations, need positive net income streams to maintain and develop their operations over the long run. Net income may increase as more hospitals implement cost-saving efficiencies. However, it is unlikely these changes will generate enough savings to quickly restore net income levels to pre-COVID levels, especially in states with minimum nurse staffing ratios, like California. This pressure will likely lead hospitals to raise prices to their commercially insured patients and their health plans. A recent study reported signs of this trend, “health care prices grew by 3.4% year over year growth rate—the fastest overall health care price growth since December 2007”.^14^ And, with the US hospital landscape increasingly dominated by large multi-hospital and health systems with increasing market power, higher prices will lead to higher health insurance premiums and reduced take-home pay for many workers in the months and years to come.

## Supplementary Material

qxad039_Supplementary_Data
