# Supplementary material for: Post-COVID trends in hospital financial performance: updated data from California paint an improved but challenging picture for hospitals and commercially insured patients
Source: Health Aff Sch. 2023 Aug 24;1(3):qxad039. doi: 10.1093/haschl/qxad039 (PMC11103726; doi:10.1093/haschl/qxad039)
Supplement: qxad039_Supplementary_Data [file qxad039_Supplementary_Data.zip › technical appendix methods ver 7 29 2023.docx]

**Supplemental Technical Appendix - Data and Methods**

**Data**

Our study analyzes publicly available data provided by the California Office of Health Care Access and Information (HCAI, formerly the Office of Statewide Health Planning and Development (OSHPD). California hospitals report financial and utilization data on both a quarterly and annual basis.

Our study uses HCAI quarterly data covering acute care hospitals that reported data to HCAI^[[1]](#endnote-1)^. Our sample focuses on acute care hospitals classified by HCAI as “comparable”. The sample covers approximately 80% of total hospital capacity in the state. Excluded from the analysis are Kaiser hospitals, which compose approximately 8% of statewide bed capacity, as well as state owned hospitals, long-term psychiatric hospitals, long-term care, and other noncomparable facilities.

The quarterly data are filed shortly after the close of each quarter, providing data on a more current, real-time basis. Quarterly data may be subject to revision when annual audited reports are filed. Because we are relying on quarterly data which are reported with the shortest lag time, some fields may be subject to revision when annual audited reports are filed. We compared historical quarterly and annual data covering earlier time periods and found that revisions to quarterly data for our selected measures, when compared to annual audited data, were quite limited, both on an aggregate basis and at the hospital level (although hospital level adjustments had a wider range).

**Variable Specification, Calculations, and Samples**

**Figure 1** Calculates quarterly totals summed across all hospitals for each quarter covering 2020-2022 compared to the totals for the same corresponding quarter in 2019 (pre-COVID). The sample includes a panel of 348 hospitals that reported data for all 16 quarters (2019-2022).

**Figure 2** Calculates average length of stay by summing total inpatient days and total inpatient admissions across all hospitals and then dividing total(summed) inpatient days by total (summed) inpatient admissions across the panel of hospitals that reported data for all quarters. The Figure presents the average length of stay for each quarter beginning in Q1 of 2018 through Q4 of 2022. In addition, the percentage change in average length of stay is calculated for each quarter beginning in Q1 of 2018 compared to the corresponding quarters of 2017.

**Table 1** uses data from HCAI from the file that reports annual totals (Sum of Four Quarters for each year 2017-2022) and for one quarter, 2023-Q1. The sample includes all comparable reporting hospitals that reported for each time-period rather than a fixed panel. This is approximately 20 more hospitals than the panel of hospitals that reported for all quarters.

**Figure 4:** Calculates the total margins for each hospital and summarizes the distribution of hospital total margins at different percentile cut points: (%), 10^th^, Median and 75^th^ Percentile Values: 2019 – 2023-Q1. The samples for this Figure are based on all hospitals reporting for each time-period. Sample sizes (N) for each time are as follows: 367, 368, 366, 363, 363.

**Variable Definitions from HCAI**

**Non-operating expense:** The expenses of a hospital which is not directly related to patient care, related patient services, or the sale of related goods. For example, non-operating expenses includes losses on sale of hospital property and retail operations expenses. Non-operating revenue represents the revenue of a hospital which is not directly related to patient care, related patient services, or the sale of related goods. For example, non-operating revenue includes unrestricted gifts, unrestricted income from endowment funds, gain on sale of hospital properties, and retail operation revenue.

**Variable Definitions from HCAI**

**Type of Hospital (Comparable):**  A hospital's report is coded as comparable or non-comparable. There are six types of hospitals: COMPARABLE-Includes hospitals whose data and operating characteristics are comparable with other hospitals, KAISER-Includes hospitals operated by Kaiser Hospital Foundation, Also includes the two regional Kaiser organization entities, which report consolidated financial data for all the hospitals in the regions., LTC Emphasis- Includes large hospitals which emphasize long-term care (LTC) services, PHF-Includes hospitals licensed as Psychiatric Healthy Facilities, which provide mental health services, SHRINERS-Includes hospitals operated by Shriners Hospitals for Children which do not charge for services provided. STATE-Includes State hospitals, which provide care to the mentally and developmentally disabled.

**Hospital Type of Care (Acute)**: Coding as follows: "General Acute"; Other Non- Acute: "Childrens", "Psychiatric" , "Specialty". Our sample includes only General Acute hospitals.

**Inpatient admission:** The formal acceptance by a health facility of a patient who is to be provided with room, board, and continuous nursing service in an area of the health facility where patients generally stay at least overnight.

**Inpatient Day:** When a person who is formally admitted to the hospital for continuous general nursing service, including room and board accommodations, in an area of the hospital where patients stay at least overnight.

**Average Length of Stay:** Total inpatient days divided by total inpatient admissions.

**Adjusted Inpatient Days:** a scaling factor to account for outpatient care volume and is calculated by dividing a facility's gross patient revenue by its gross inpatient revenue and multiplying by total inpatient days.

**Net Patient Revenue:** The amount of aggregate money generated from patient services that is collected from payors, including all payors private insurance, Medicaid and Medicare and other payors. Calculated as gross patient revenue less deductions from revenue. This amount is more comparable than gross patient revenue because it indicates the actual amount received from patients and third party payers. Includes disproportionate share payments (before any transfers to related entities) and capitation premium revenue.

**Other Operating Revenue:** Revenue generated by health care operations from non-patient care services to patients and others. Examples include non-patient food sales, refunds and rebates, supplies sold to non-patients, and Medical Records abstract sales. Does not include interest income.

**Total Operating Revenue;** Total revenue derived from sources related to a hospital's everyday patient services and patient related business operations. Operating Revenue includes revenue directly related to the rendering of patient care services and revenue from non-patient care services to patients and sales and activities to persons other than patients

**Non-Operating Revenue:** Revenue generated by activities of an enterprise, usually not associated with patient services. The revenue of a hospital which is not directly related to patient care, related patient services, or the sale of related goods. For example, non-operating revenue includes unrestricted gifts, unrestricted income from endowment funds, gain on sale of hospital properties, and retail operation revenue.

**Operating Expense:** An expense incurred in conducting the ordinary major activities of an enterprise, usually excluding "nonoperating" expense or income deductions.

**Pre-Tax Net Income:** Financial calculation determined by adding Net Patient Revenue + Other Patient Revenue – Total Operating Expenses + Net Non-operating Revenue and Expenses

**Net income from Operations:** Includes net patient revenue plus other operating revenue minus operating expenses. Net income from non-operating revenue is non-operating revenue minus non-operating expense. Total net income is calculated as total revenue from all sources (operating and non-operating) minus total expenses (operating and non-operating).

**Non-Operating cost (or expense):** An expense not incurred in conducting the ordinary major activities of an enterprise, usually associated with generating "nonoperating" revenue.

**Pre-Tax Net Income:** Financial calculation determined by adding Net Patient Revenue + Other Patient Revenue – Total Operating Expenses + Net Non-operating Revenue and Expenses

**Operating (Net Income) Margin:** Financial calculation determined by Net from Operations divided by Total Operating Revenue then times the total by 100.

**Total (Net Income) Margin:** Financial calculation determined by Pre-Tax Net Income divided by Total Operating Revenue then times the total by 100.

1. https://data.chhs.ca.gov [↑](#endnote-ref-1)
